# A practical guide for using lithium halocarbenoids in homologation reactions

**DOI:** 10.1007/s00706-018-2232-9

**Published:** 2018-06-11

**Authors:** Serena Monticelli, Marta Rui, Laura Castoldi, Giada Missere, Vittorio Pace

**Affiliations:** Department of Pharmaceutical Chemistry, University of Vienna, Vienna, Austria

**Keywords:** Lithiation, Carbenoids, Organometallics

## Abstract

**Abstract:**

Lithium halocarbenoids are versatile reagents for accomplishing homologation processes. The fast α-elimination they suffer has been considered an important limitation for their extensive use. Herein, we present a series of practical considerations for an effective employment in the homologation of selected carbon electrophiles.

**Graphical abstract:**

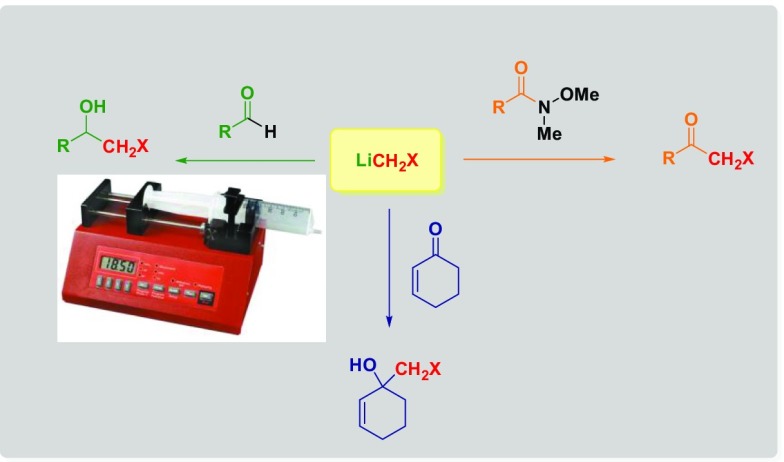

**Electronic supplementary material:**

The online version of this article (10.1007/s00706-018-2232-9) contains supplementary material, which is available to authorized users.

## Introduction

Methylenating agents are recognized as valuable synthetic tools in homologation reactions, allowing the formal insertion of a methylene unit (i.e., CH_2_) into a given preformed bond. Classical examples of homologation processes are represented by the carbon chain extension or the ring expansion of carbonyl compounds [1, 2].

Carbenoidic reagents play a prominent role within the plethora of homologating agents [3–6]. The term carbenoid was introduced by the pioneers in the field Closs and Moss who defined their chemical reactivity “qualitatively analogous to those of carbenes without necessarily being free divalent carbon species” [7]. Accordingly, organometallic compounds containing a metal atom (e.g., Li, Mg) and, at least one electronegative element (e.g., halogen) linked to the same carbon, have been referred to as carbenoids, thus considering their carbene-like features [8].

A significant advancement in the field originated from the work of Gert Köbrich and coworkers in the 1960s [9]. These milestones still represent the key concepts in carbenoid chemistry and put the bases for the rational design and understanding of reactions involving these versatile synthetic tools. The concomitant presence of an electron-donating and electron-withdrawing substituent at the carbon center determines the so-called ambiphilicity of these reagents [5, 10]. Thus, carbenoids display a dual reactivity ranging from nucleophilic to electrophilic [6, 11, 12]. Depending on the experimental conditions, they may selectively exhibit only one of these two properties [13–17]: it is normally accepted that the nucleophilic behavior is shown at low temperatures, while their electrophilicity comes into play at higher temperatures (Scheme 1) [6, 18, 19]. This key characteristic of carbenoid reagents can be explained taking into consideration structures, which in principle can provide two different ionization forms. On the one hand, a negative charge is localized at the carbon atom (i.e., it becomes nucleophilic), while in the other case the carbon atom brings a positive charge (i.e., it becomes electrophilic).

**Figure:**
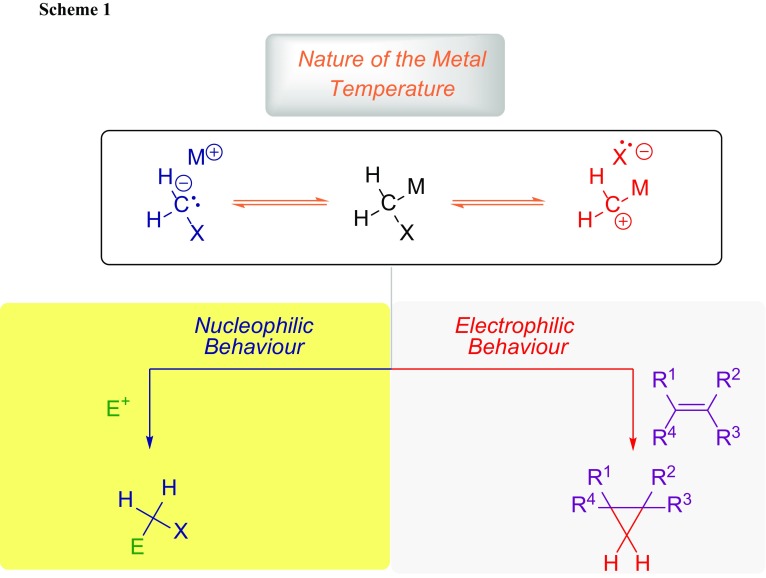

Given these premises, one may individuate two different reactions categories in which carbenoids are involved: (1) nucleophilic additions (eventually followed by elimination); (2) cyclopropanation-type processes (Simmons–Smith like chemistry) [20, 21]. It is important to stress that carbenoids of lithium and magnesium, because of their excellent nucleophilicity, do react predominantly as carbanions [6, 13–16]. On the other hand, less nucleophilic carbenoids such as zinc or rhodium linked ones exhibit preferentially an electrophilic behavior [4, 22].

In recent years, our group launched a research program [23] focused on the use of carbenoid-type reagents for the homologation of different carbon (Weinreb amides [24–32], ketones [33, 34], isocyanates [35–38]) or heteroatom electrophiles [39] for preparing in a single step α-halo or rearranged (thereof) derivatives [40]. We observed a paramount importance of the conditions employed for generating the carbenoid and, herein, we disclose full details on how to prepare and use these highly reactive species under Barbier type conditions [41, 42].

## Results and discussion

We evaluated the employment of a syringe pump, as a practical tool to modulate the addition rate of organolithium and its influence in carbenoid-mediated homologation reactions. A straightforward strategy to yield halohydrins requires the treatment of an aldehyde or a ketone with halocarbenoids. Reactions involving carbenoids need an excess of both halomethyl precursor and Li-source to overcome the limiting instability after their generation at − 78 °C [18]. The carbenoid species were generated in situ, by adding MeLi–LiBr (2.8 equiv)—using an automatic syringe pump—to a solution of ICH_2_Cl (3.0 equiv) and electrophile (1.0 equiv). Accordingly, we firstly evaluated the reproducibility of the reaction (reported by Matteson in 1986) [43] on benzaldehyde (**1**) being the substrate endowed with an excellent electrophilic profile. Moreover, for comparative purposes, we performed an exploratory reaction, adopting a manual addition of the organolithium reagent (MeLi–LiBr), at − 78 °C. The synthetic protocol led us to obtain the desired chlorohydrin **2a**, in relatively low yield (54%). Considering this result, we directed our efforts towards the identification of the optimal conditions to achieve a complete conversion of benzaldehyde into the corresponding **2a**, exploiting a syringe pump. Different temperatures—ranging from − 78 to 20 °C—were screened to evaluate the conversion of the aldehyde into the desired product and, the subsequent generation and distribution of side-products (i.e., epoxide **3** or alcohol **4**). The so-obtained chloromethyllithium promptly reacts with the aldehyde present in the reaction environment affording chlorohydrin **2a**.

LiCH_2_Cl-mediated homologations show the best compromise between stability and reactivity at − 78 °C. Nevertheless, the syringe pump-mediated addition of the lithium reagent allows with a rate of 0.200 cm^3^/min to increase the reaction temperature up to − 15 °C (Table 1, entries 1–6 and 8), obtaining the corresponding homologated product **2a** in good yield. Conversely, increasing the temperature from − 5 to 20 °C (Table 1, entries 9–11), the homologated product **2a** is gradually converted into epoxide **3** via an internal S_N_2 reaction and the formation of **4** is increased due to the competitive attack of MeLi to the carbonyl [44]. Increasing the rate from 0.200 to 0.400 cm^3^/min (Table 1, entry 7) at − 25 °C resulted in an excellent conversion into **2a** and no formation of **4** was detected. As the addition rate of MeLi–LiBr was reduced to 0.050 cm^3^/min at 20 °C (Table 1, entry 12), we observed a higher conversion of **2a** into epoxide **3**, and reduced attack of MeLi to the aldehyde. The results obtained can be translated to a higher control for generating the Li-carbenoid species at elevated temperatures as well as maintaining a good stability and reactivity. In turn, for this specific case, the thermal instability of halohydrin **2a** lies on the boundary of − 25 to − 15 °C.

**Table 1:** Controlled generation of Li-carbenoid, LiCH_2_Cl

Entry	*T*/°C	**2a**/%	**3**/%	**4**/%
1^a^	− 78	94	0	0
2^a^	− 65	94	0	0
3^a^	− 55	94	0	3
4^a^	− 45	95	0	2
5^a^	− 35	88	0	3
6^a^	− 25	85	0	5
7^b^	− 25	93	0	0
8^a^	− 15	89	0	4
9^a^	− 5	65	8	6
10^a^	0	68	10	9
11^a^	20	25	49	20
12^c^	20	26	60	4

^a^0.200 cm^3^/min drop rate of MeLi–LiBr

^b^0.400 cm^3^/min drop rate of MeLi–LiBr

^c^0.050 cm^3^/min drop rate of MeLi–LiBr

We then studied the effect of temperature on the reactivity of halocarbenoids generated by different halomethyl sources to compare the behavior of Li-carbenoid species.

The use of diiodomethane for generating iodomethyllithium showed good results under the reaction condition below − 55 °C (Table 2, entries 1–3). The increase of the temperature favored the formation of the corresponding epoxide **3**. Notably, compound **4** does not exceed 18% even at 0 °C (Table 2, entries 7–9).

**Table 2:** Temperature dependency of different halomethyl carbenoids

Entry	*T*/°C	CH_2_I_2_			CH_2_Br_2_			ICH_2_Br		
		**2b**/%	**3**/%	**4**/%	**2c**/%	**3**/%	**4**/%	**2c**/%	**3**/%	**4**/%
1	− 78	90	3	2	78	0	5	78	0	0
2	− 65	81	2	12	62	0	24	79	4	3
3	− 55	86	3	5	71	0	19	84	3	5
4	− 45	68	24	4	90	0	5	81	7	3
5	− 35	45	35	11	85	0	7	87	7	2
6	− 25	45	41	5	39	5	48	64	28	3
7	− 15	32	39	18	46	10	34	52	40	3
8	− 5	22	54	4	0	20	68	34	12	51
9	0	23	54	10	4	16	66	0	16	75

Difficulties in controlling the generation of bromomethyllithium carbenoid arose when using CH_2_Br_2_ as dihalomethane. Evidently, the Li-carbenoid is generated at a minor extent in comparison with ICH_2_Cl and the reaction is dominated by a direct nucleophilic addition of MeLi on carbonyl at temperature up to − 25 °C (Table 2, entries 6–9). ICH_2_Br was then used as alternative bromomethyl source, showing similar results to ICH_2_Cl, albeit in slightly lower conversion into bromohydrin **2c**. It showed a good control in generating the bromomethyllithium carbenoid and maintaining a good reactivity until − 35 °C (Table 2, entry 5). Increasing the temperature, resulted in the bromohydrin ring closure to afford epoxide **3** (Table 2, entry 7) and at 0 °C no bromohydrin **2** was anymore detected (Table 2, entry 9). At higher temperature, MeLi–LiBr possesses a higher reactivity towards benzaldehyde than ICH_2_Br; in fact compound **4** represents the main reaction product at 0 °C (Table 2, entry 9). In the light of these data, ICH_2_Cl remains the best source for generating and maintaining a good reactivity of the Li-carbenoid, LiCH_2_Cl, towards benzaldehyde.

With the aim to widen the stability/reactivity study employing syringe pump, other electrophiles were subjected to the previous reaction conditions. 2-Phenylacetaldehyde (**5**), phenyl Weinreb amide (**6**), and cyclohexenone (**7**) were selected for this scope.

Extending the chain with one carbon atom, 2-phenylacetaldehyde resulted in a quasi-stable conversion towards **5a** although in a minor consent when compared to benzaldehyde **1**.

Afterwards, the homologation of Weinreb amides—a class of acylating agents particularly suited for α-substituted organolithium reagents [45–48]—was evaluated. *N*-Methoxy-*N*-methylbenzamide (**6**) showed rather good results until − 45 °C, where the conversion starts to decrease, however, maintaining 21% conversion at 0 °C (Table 3, entries 5–9).

**Table 3:** Study of ICH_2_Cl reactivity toward electrophiles at different temperature

Entry	*T*/°C	**5a**	**6a**	**7a**
1	− 78	64	82	93
2	− 65	65	74	95
3	− 55	54	72	97
4	− 45	68	69	97
5	− 35	68	59	94
6	− 25	70	51	91
7	− 15	57	33	85
8	− 5	40	38	59
9	0	44	21	59

Numbers signify conversion (%) of **5**, **6**, and **7** towards their corresponding homologated product **5a**, **6a**, **7a** based on ^1^H NMR calculations

The α,β-unsaturated cyclic ketone **7** was then chosen as electrophile, due to our previous interest in its challenging reactivity [40]. Surprisingly, it showed a very good stability profile even at temperature close to 0 °C and practically the same reactivity of benzaldehyde with chloromethyllithium (Table 3, entries 5–9). To reach full conversion of cyclohexenone into chlorohydrin **7a**, different additives were tested. They could promote the formation and improve the stability of the Li-carbenoid and ultimately increase the electrophilicity of the cyclohexenone.

**Fig. 1:**
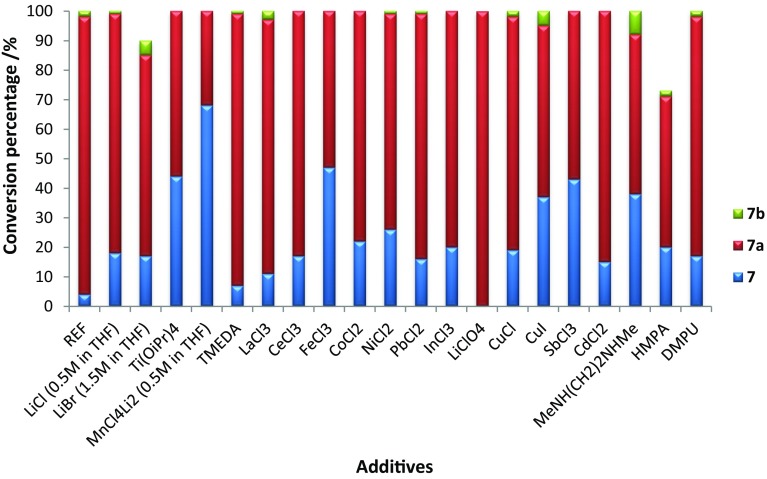
Use of additives/salts

As shown in Table 4 and in Fig. 1, the reference conditions were set at − 35 °C for 1 h, upon which small conversion into aldehyde **7b** (as a consequence of the Meinwald rearrangement) was started to be observed [40].

**Table 4:** Use of additives/salts

Entry	Additive	*T*/°C	**7**	**7a**	**7b**
REF	REF	− 35	4	94	2
1	LiCl (0.5 M in THF)	− 35	18	81	1
2	LiBr (1.5 M in THF)	− 35	17	68	5
3	Ti(O*i*Pr)_4_	− 35	44	56	0
4	MnCl_4_Li_2_ (0.5 M in THF)	− 35	68	32	0
5	TMEDA	− 35	7	92	1
6	LaCl_3_	− 35	11	86	3
7	CeCl_3_	− 35	17	83	0
8	FeCl_3_	− 35	47	53	0
9	CoCl_2_	− 35	22	78	0
10	NiCl_2_	− 35	26	73	1
11	PbCl_2_	− 35	16	83	1
12	InCl_3_	− 35	20	80	0
13	LiClO_4_	− 35	0	> 99	0
14	CuCl	− 35	19	79	2
15	CuI	− 35	37	58	5
16	SbCl_3_	− 35	43	57	0
17	CdCl_2_	− 35	15	85	0
18	MeNH(CH_2_)_2_NHMe	− 35	38	54	8
19	HMPA	− 35	20	51	2
20	DMPU	− 35	17	81	2

Numbers signify conversion (%) of **7** to **7a** and **7b** based on ^1^H NMR calculations

From the obtained results, we can conclude that the addition of additives has almost no beneficiary effect on the conversion towards **7a**. Nevertheless, there are a few cases worth mentioning. Although entries 2, 15, and 18 (Table 4, Fig. 1) show a decrease in the homologated product **7a**; they also resulted in a slightly higher conversion into the corresponding aldehyde **7b**. Surprisingly, full conversion of cyclohexenone **7** into **7a** was observed only when lithium perchlorate LiClO_4_ (Table 4, entry 15; Fig. 1) was used. With this result in hand, we examined the concentration dependency of cyclohexenone in combination with LiClO_4_. As reported in Table 5, the optimal concentration (Table 5, entry 4) was found to be 1 M and it represents the concentration used in all the previous experiments.

**Table 5:** Concentration dependency in the homologation of **7** towards **7a**

Entry	*c*/M	**7**	**7a**	**7b**
1	0.01	36	62	1
2	0.1	39	58	3
3	0.5	14	84	2
4	1	1	99	0
5	2	35	63	2
6	10	33	64	3

Numbers signify conversion (%) of **7** to **7a** and **7b** based on ^1^H NMR calculations

## Conclusions

The well-known instability of lithium halocarbenoids has represented a significant challenge for their employment in synthesis [49]. Despite the usefulness, the requirement for strict conditions for counterbalancing the degradative α-elimination had somehow constituted the main limitation, thus obscuring the innate potential. In this study, we identified the ideal conditions (stoichiometry, temperature, syringe pump) for finely tuning their generation and reactivity towards common carbon electrophiles.

## Electronic supplementary material

Below is the link to the electronic supplementary material.

Supplementary material 1 (PDF 1660 kb)
