# Supplementary material for: A practical guide for using lithium halocarbenoids in homologation reactions
Source: Monatsh Chem. 2018 Jun 11;149(7):1285–91. doi: 10.1007/s00706-018-2232-9 (PMC6006224; doi:10.1007/s00706-018-2232-9)
Supplement: Supplementary file 1 — Supplementary material 1 (PDF 1660 kb) [file 706_2018_2232_MOESM1_ESM.pdf]

Supporting Information

A Practical Guide for Using Lithium Halocarbenoids in Homologation Reactions

Serena Monticelli ● Marta Rui ● Laura Castoldi ● Giada Missere ●Vittorio Pace✉

Department of Pharmaceutical Chemistry, University of Vienna, Vienna, Austria

✉*Vittorio Pace*
vittorio.pace@univie.ac.at

Table of contents 1

Materials and Methods 2

General Procedures for the Chemoselective Addition of Lithium-carbenoids 2

Copies of NMR spectra 7

Materials and methods

All ^{1}H NMR and ^{13}C NMR spectra were recorded on Bruker Avance spectrometers operating at 200, 300, 400 or 500 MHz and at 50, 75, 100, or 125 MHz, respectively, from $CDCl_3$ solutions. The (residual) solvent signal was used as an internal standard which was related to TMS with δ 7.26 ppm (^{1}H) and δ 77.0 ppm (^{13}C). Spin-spin coupling constants (*J*) are given in Hz. In some cases, full and unambiguous assignment of all ^{1}H, ^{13}C, resonances was performed by combined application of standard NMR techniques, such as APT, DEPT, HSQC, HMBC and NOESY experiments.

All melting points are uncorrected. Column chromatography purifications were conducted on silica gel 60 (40-63 μm). TLC was carried out on aluminum sheets pre-coated with silica gel 60F254; the spots were visualized under UV light (λ = 254 nm) and/or $KMnO_4$ (aq.) was used as revealing system.

Starting materials were supplied from commercial sources otherwise indicated.

Elementary microanalyses were carried out using a Leco® CHNS 932 equipment.

General Procedures for the Chemoselective Addition of Lithium-carbenoids

2-chloro-1-phenylethan-1-ol (2a)

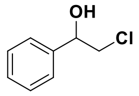

2a

To a solution of aldehyde **2** (200 mg, 1.88 mmol, 1.0 equiv), in THF (1.9 mL) cooled at -78 °C was added freshly distilled chloroiodomethane (997 mg, 0.41 mL, 5.65 mmol, 3.0 equiv), followed by the addition of a solution of MeLi-LiBr complex (1.5 M, 3.52 mL, 5.28 mmol, 2.8 equiv) operated by syringe pump with flow of 0.2 ml/min. The mixture was stirred for 1 h before it was quenched with saturated aq. NH_4Cl (2 mL) and extracted with Et_2O (3 x 5 mL). The organic layer was washed with brine (5 mL), dried over Na_2SO_4, filtered and solvent was removed under reduced pressure to give a crude which after chromatographic purification on silica gel (eluent hexane/ethyl acetate 4:1, v/v) afforded chlorohydrin **2a** (276 mg, 94% yield).

^{1}H NMR (200 MHz, $CDCl_3$) δ: 7.38 (m, 5H, Ph), 4.81 (dd, *J*=8.5, 3.7 Hz, 1H, CH), 3.75-3.46 (m, 2H, CH_2), 2.74 (brs, 1H, OH).

^{13}C NMR (50 MHz, $CDCl_3$) δ: 139.9 (Ph C-1), 128.6 (Ph C-3,5), 128.4 (Ph C-4), 126.0 (Ph C2,6), 74.0 (CH), 50.7 (CH_2).

HRMS (ESI), *m/z*: calcd. For C_8H_9ClNaO 179.0234 [M+Na]; found 179.0238.

2-iodo-1-phenylethan-1-ol (2b)

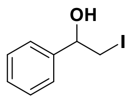

2b

To a solution of aldehyde **2** (200 mg, 1.88 mmol, 1.0 equiv), in THF (1.9 mL) cooled at -78 °C was added diiodomethane (1514 mg, 0.46 mL, 5.65 mmol, 3.0 equiv), followed by the addition of a solution of MeLi-LiBr complex (1.5 M, 3.52 mL, 5.28 mmol, 2.8 equiv) operated by syringe pump with flow of 0.2 ml/min. The mixture was stirred for 1 h before it was quenched with saturated aq. NH_4Cl (2 mL) and extracted with Et_2O (3 x 5 mL). The organic layer was washed with brine (5 mL), dried over Na_2SO_4, filtered and solvent was removed under reduced pressure to give a crude which after chromatographic purification on silica gel (eluent hexane/ethyl acetate 4:1, v/v) afforded iodohydrin **2b** (420 mg, 90% yield).

^{1}H NMR (200 MHz, $CDCl_3$) δ: 7.37 (m, 5H, Ph), 4.84 (dd, *J*=8.6, 3.8 Hz, 1H, CH), 3.59-3.31 (m, 2H, CH_2), 2.44 (brs, 1H, OH).

^{13}C NMR (50 MHz, $CDCl_3$) δ: 141.1 (Ph C-1), 128.7 (Ph C-3,5), 128.4 (Ph C-4), 125.7 (Ph C2,6), 74.1 (CH), 15.4 (CH_2).

HRMS (ESI), *m/z*: calcd. For C_8H_9INaO 270.9590 [M+Na]; found 270.9591.

2-bromo-1-phenylethan-1-ol (2c)

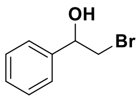

2c

Procedure A:

To a solution of aldehyde **2** (200 mg, 1.88 mmol, 1.0 equiv), in THF (1.9 mL) cooled at -78 °C was added bromoiodomethane (1247 mg, 0.43 mL, 5.65 mmol, 3.0 equiv), followed by the addition of a solution of MeLi-LiBr complex (1.5 M, 3.52 mL, 5.28 mmol, 2.8 equiv) operated by syringe pump with flow of 0.2 ml/min. The mixture was stirred for 1 h before it was quenched with saturated aq. NH_4Cl (2 mL) and extracted with Et_2O (3 x 5 mL). The organic layer was washed with brine (5 mL), dried over Na_2SO_4, filtered and solvent was removed under reduced pressure to give a crude which after chromatographic purification on silica gel (eluent hexane/ethyl acetate 4:1, v/v) afforded bromohydrin **2b** (294 mg, 78% yield).

Procedure B:

To a solution of aldehyde **2** (200 mg, 1.88 mmol, 1.0 equiv), in THF (1.9 mL) cooled at -78 °C was added dibromomethane (983 mg, 0.40 mL, 5.65 mmol, 3.0 equiv), followed by the addition of a solution of MeLi-LiBr complex (1.5 M, 3.52 mL, 5.28 mmol, 2.8 equiv) operated by syringe pump with flow of 0.2 ml/min. The mixture was stirred for 1 h before it was quenched with saturated aq. NH_4Cl (2 mL) and extracted with Et_2O (3 x 5 mL). The organic layer was washed with brine (5 mL), dried over Na_2SO_4, filtered and solvent was removed under reduced pressure to give a crude which after chromatographic

purification on silica gel (eluent hexane/ethyl acetate 4:1, v/v) afforded bromohydrin **2b** (294 mg, 78% yield).

^{1}H NMR (200 MHz, CDCl$_3$) δ: 7.37 (m, 5H, Ph), 4.84 (dd, *J*=8.6, 3.8 Hz, 1H, CH), 3.59-3.31 (m, 2H, CH$_2$), 2.44 (brs, 1H, OH).

^{13}C NMR (50 MHz, CDCl$_3$) δ: 141.1 (Ph C-1), 128.7 (Ph C-3,5), 128.4 (Ph C-4), 125.7 (Ph C2,6), 74.1 (CH), 15.4 (CH$_2$).

HRMS (ESI), *m/z*: calcd. For C_8H_9BrNaO 222.9729 [M+Na]; found 222.9730.

2-phenyloxirane (3)

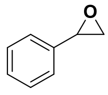

3

^{1}H NMR (400 MHz, CDCl$_3$) δ: 7.33 (m, 5H, Ph), 3.86 (dd, 1H, CH), 3.15 (dd, 1H, CH), 2.81 (dd, 1H, CH).

^{13}C NMR (100 MHz, CDCl$_3$) δ: 137.5 (Ph C-1), 128.3 (Ph C-4), 128.0 (Ph C2,6), 125.3 (Ph C-3,5), 52.1 (CH), 51.0 (CH$_2$).

1-phenylethan-1-ol (4)

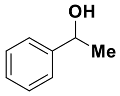

4

^{1}H NMR (400 MHz, CDCl$_3$) δ: 7.23-7.47 (m, 5H, Ph), 4.90 (q, *J*=6.5 Hz, 1H, CH), 1.93 (brs, 1H, OH), 1.50 (d, *J*=6. 4 Hz, 1H, CH$_3$).

^{13}C NMR (100 MHz, CDCl$_3$) δ: 145.8 (Ph C-1), 128.5 (Ph C-4), 127.4 (Ph C2,6), 125.4 (Ph C-3,5), 70.4 (CH), 25.1 (CH$_3$).

1-chloro-3-phenylpropan-2-ol (5a)

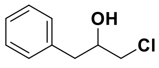

5a

To a solution of aldehyde **5** (200 mg, 1.66 mmol, 1.0 equiv), in THF (1.9 mL) cooled at -78 °C was added freshly distilled chloroiodomethane (881 mg, 0.36 mL, 5.0 mmol, 3.0 equiv), followed by the addition of a solution of MeLi-LiBr complex (1.5 M, 3.11 mL, 4.66 mmol, 2.8 equiv) operated by syringe pump with flow of 0.2 ml/min. The mixture was stirred for 1 h before it was quenched with saturated aq. NH_4Cl (2 mL) and extracted with Et_2O (3 x 5 mL). The organic layer was washed with brine (5 mL), dried over Na_2SO_4, filtered and solvent was removed under reduced pressure to give a crude which after chromatographic purification on silica gel (eluent hexane/ethyl acetate 4:1, v/v) afforded chlorohydrin **2a** (180 mg, 64% yield).

^{1}H NMR (200 MHz, CDCl$_3$) δ: 7.14-7.30 (m, 5H, Ph), 3.99 (m, 1H, CH), 3.38-3.59 (m, 2H, CH_2Cl), 2.82 (d, 2H, CH_2Ph), 2.09 (brd, 1H, OH).

^{13}C NMR (50 MHz, $CDCl_3$) δ: 137.0 (Ph C-1), 129.3 (Ph C-3,5), 128.7 (Ph C2,6), 126.8 (Ph C-4), 72.2 (CH), 49.2 (CH_2Cl), 40.6 (CH_2Ph).

2-chloro-1-phenylethan-1-one (6a)

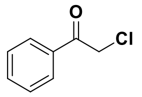

6a

To a solution of Weinreb amide **6** (200 mg, 1.21 mmol, 1.0 equiv), in THF (1.2 mL) cooled at -78 °C was added freshly distilled chloroiodomethane (641 mg, 0.26 mL, 3.63 mmol, 3.0 equiv), followed by the addition of a solution of MeLi-LiBr complex (1.5 M, 2.26 mL, 3.39 mmol, 2.8 equiv) operated by syringe pump with flow of 0.2 ml/min. The mixture was stirred for 1 h before it was quenched with saturated aq. NH_4Cl (2 mL) and extracted with Et_2O (3 x 5 mL). The organic layer was washed with brine (5 mL), dried over Na_2SO_4, filtered and solvent was removed under reduced pressure to give a crude which after chromatographic purification on silica gel (eluent hexane/ethyl acetate 4:1, v/v) afforded chloroketone **6a** (153 mg, 82% yield).

^{1}H NMR (400 MHz, $CDCl_3$) δ: 7.92 (m, 2H, Ph H-2,6), 7.57 (m, 1H, Ph H-4), 7.47 (m, 2H, Ph H-3,5), 4.70 (s, 2H, CH_2).

^{13}C NMR (100 MHz, $CDCl_3$) δ: 190.8 (C=O), 134.0 (Ph C-1), 133.7 (Ph C-4), 128.6 (Ph C2,6), 128.2 (Ph C-3,5), 46.0 (CH_2).

IR (NaCl, νmax, cm^{-1}): 3062, 1691, 1589.

Elemental Analysis (%) for C_8H_7ClO. Calcd: C, 62.16; H, 4.56. Found: C, 62.31; H, 4.72.

1-(chloromethyl)cyclohex-2-en-1-ol (7a)

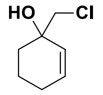

7a

Procedure A:

To a solution of ketone **7** (200 mg, 2.08 mmol, 1.0 equiv) in THF (2 mL) cooled at -78 °C was added freshly distilled chloroiodomethane (997 mg, 0.41 mL, 5.65 mmol, 3.0 equiv), followed by the addition of a solution of MeLi-LiBr complex (1.5 M, 3.52 mL, 5.28 mmol, 2.8 equiv) operated by syringe pump with flow of 0.2 ml/min. The mixture was stirred for 1 hour before it was quenched with saturated aq. NH_4Cl (2 mL) and extracted with Et_2O (3 x 5 mL). The organic layer was washed with brine (5 mL),

dried over Na_2SO_4, filtered and after removing the solvent under reduced pressure, analytically pure chlorohydrin **7a** (260 mg, 85% yield) was obtained as a colourless oil.

Procedure B:

To a solution of ketone **7** (200 mg, 2.08 mmol, 1.0 equiv) and an additive (3.0 equiv) in THF (2 mL) cooled at -35 °C was added freshly distilled chloroiodomethane (997 mg, 0.41 mL, 5.65 mmol, 3.0 equiv), followed by the addition of a solution of MeLi-LiBr complex (1.5 M, 3.52 mL, 5.28 mmol, 2.8 equiv) operated by syringe pump with flow of 0.2 ml/min. The mixture was stirred for 1 hour before it was quenched with saturated aq. NH_4Cl (2 mL) and extracted with Et_2O (3 x 5 mL). The organic layer was washed with brine (5 mL), dried over Na_2SO_4, filtered and after removing the solvent under reduced pressure, analytically pure chlorohydrin **7a** was obtained as a colourless oil.

^{1} H NMR (300 MHz, $CDCl_3$) δ: 5.94 (dt, J = 10.2, 3.7 Hz, 1H), 5.64 (dt, J = 10.1, 2.3 Hz, 1H), 3.55 (d, J = 2.3 Hz, 2H), 2.21 (s, 1H), 2.15 – 1.42 (m, 7H).

^{13}C NMR (75 MHz, $CDCl_3$) δ: 132.6, 128.5, 69.1, 53.8, 33.3, 25.2, 18.8.

Elemental Analysis (%) for $C_7H_{11}ClO$. Calcd: C, 57.35; H, 7.56. Found: C, 57.51; H, 7.70.

1-methyl-2-cyclohexene-1-carbaldehyde (7b)

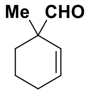

7b

^{1}H NMR (400 MHz, C_6D_6) δ: 9.24 (s, 1H, CHO), 5.67 (td, $^3J_{H3,H2}$ = 10.0 Hz, $^3J_{H3,H4}$ = 3.8 Hz, 1H, H-3), 5.26 (td, $^3J_{H2,H3}$ = 10.0 Hz, $^4J_{H2,H4}$ = 2.1 Hz, 1H, H-2), 1.74 (m, 1H, H-6), 1.68 – 1.63 (m, 2H, H-4), 1.44 – 1.26 (m, 2H, H-5), 1.07 (m, 1H, H-6), 0.87 (s, 3H, 1-CH_3).

^{13}C NMR (100 MHz, C_6D_6) δ: 201.7 (CHO), 130.7 (C-3), 128.2 (C-2), 47.5 (C-1), 30.0 (C-6), 24.9 (C-4), 22.1 (1-CH3), 19.1 (C-5).

HRMS (ESI), m/z: calcd. for $C_8H_{13}O^+$: 125.0961 $[M+H]^+$; found: 125.0964.

Copies of NMR spectra

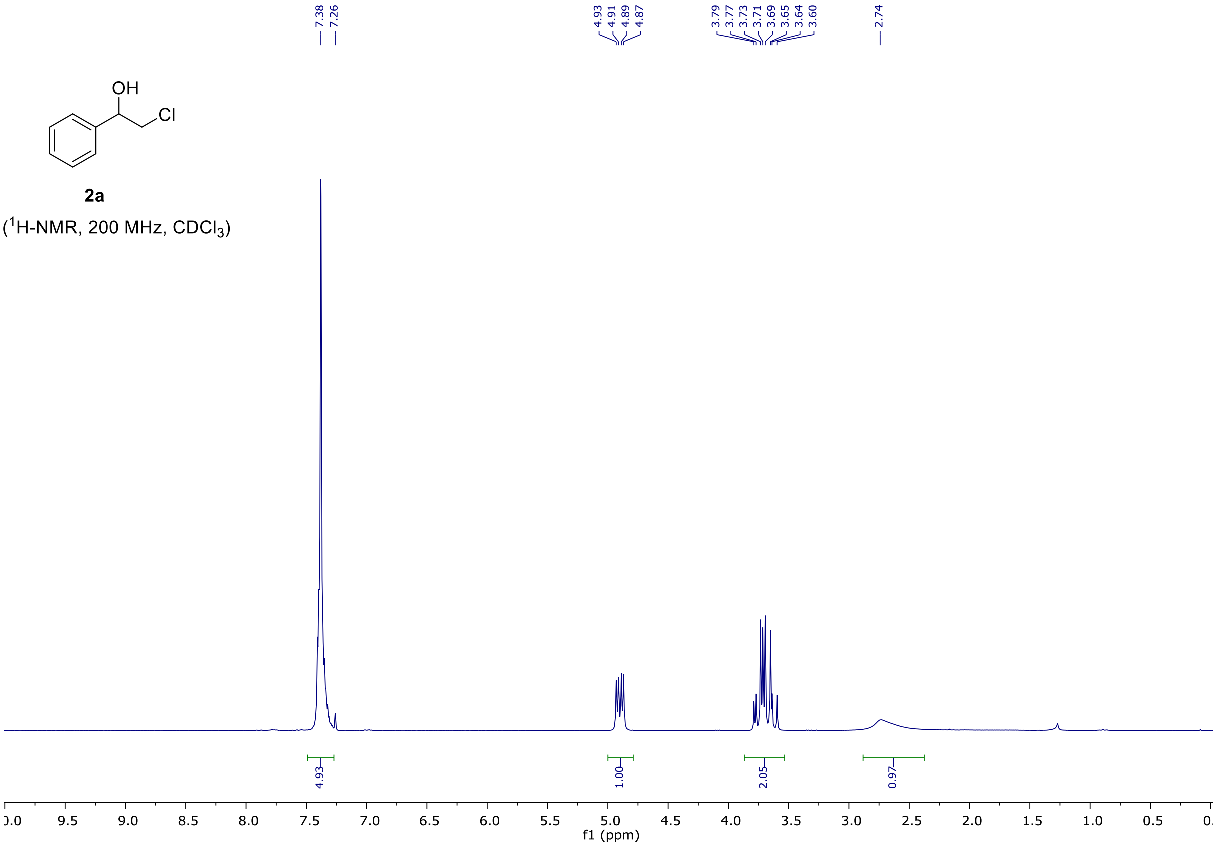

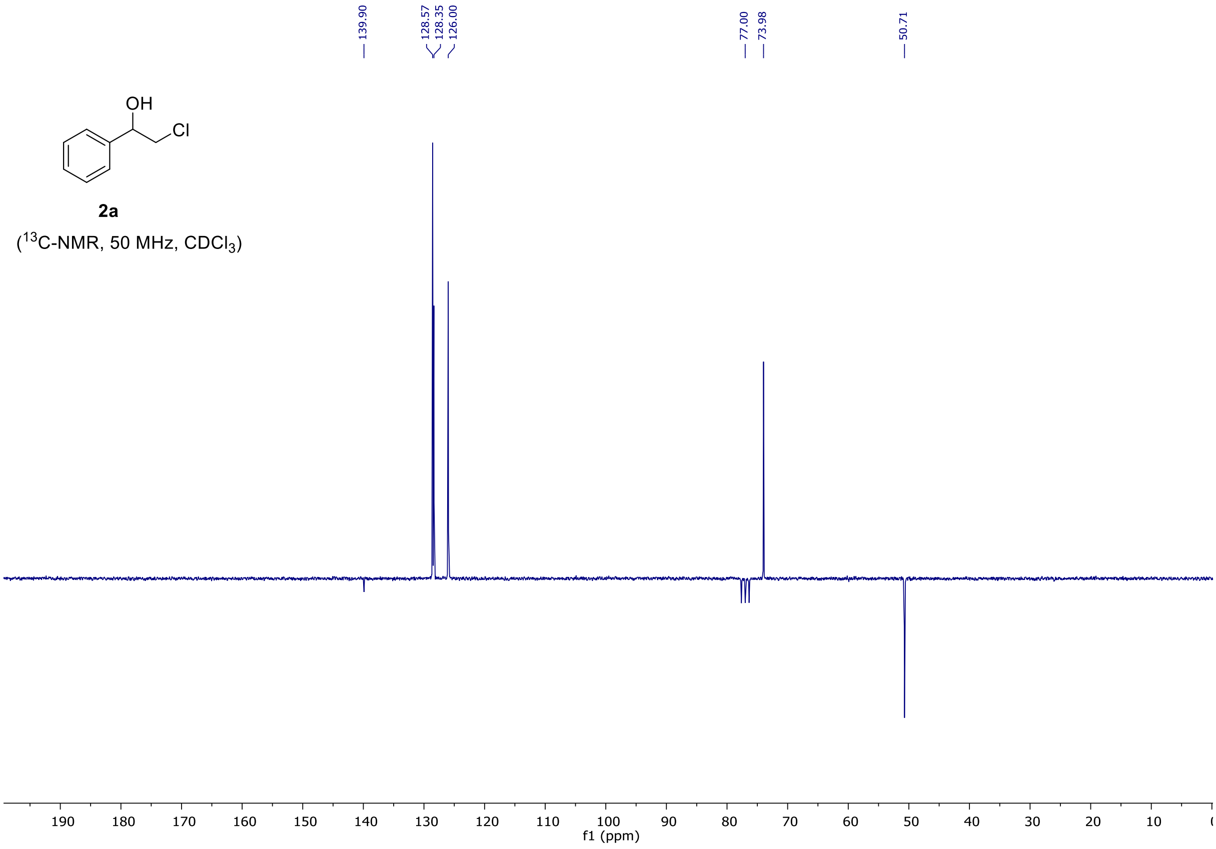

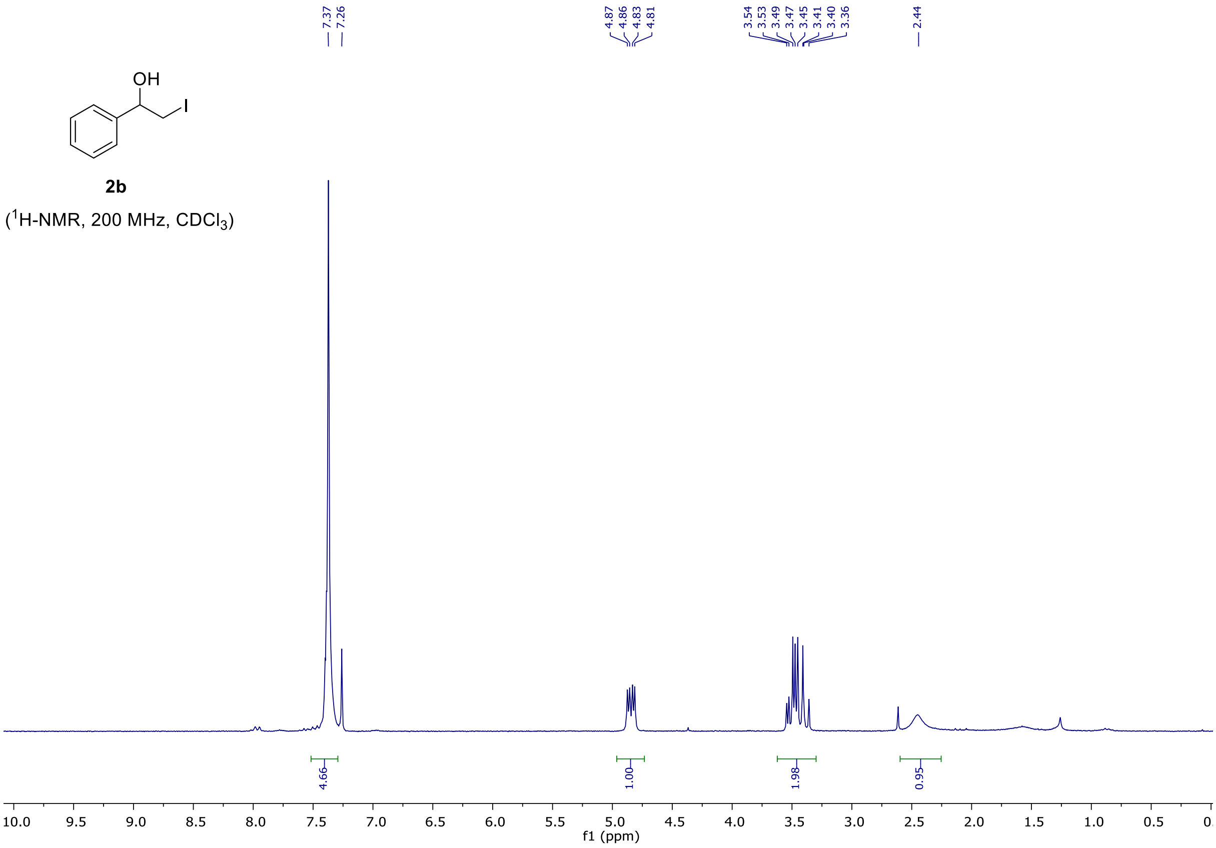

2b

(^{1}H-NMR, 200 MHz, $CDCl_3$)

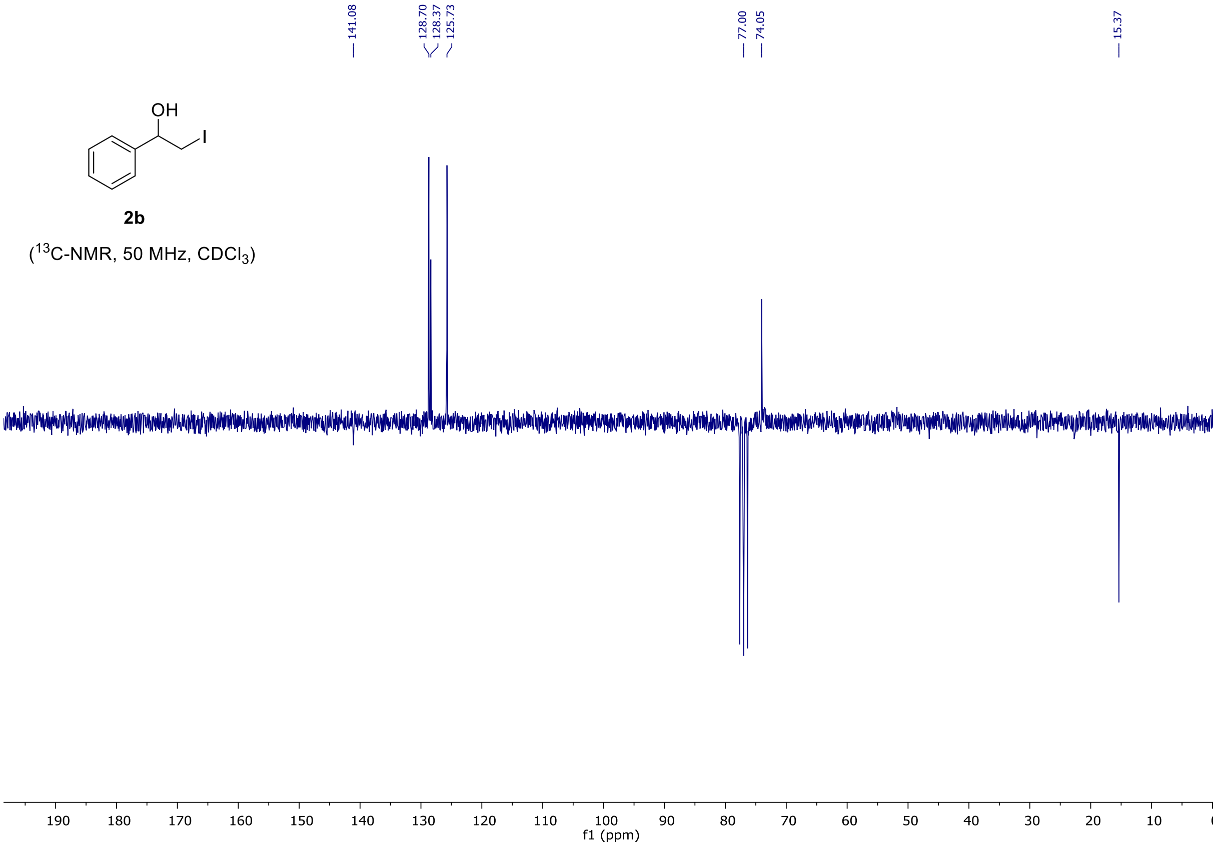

2b

(^{13}C-NMR, 50 MHz, $CDCl_3$)

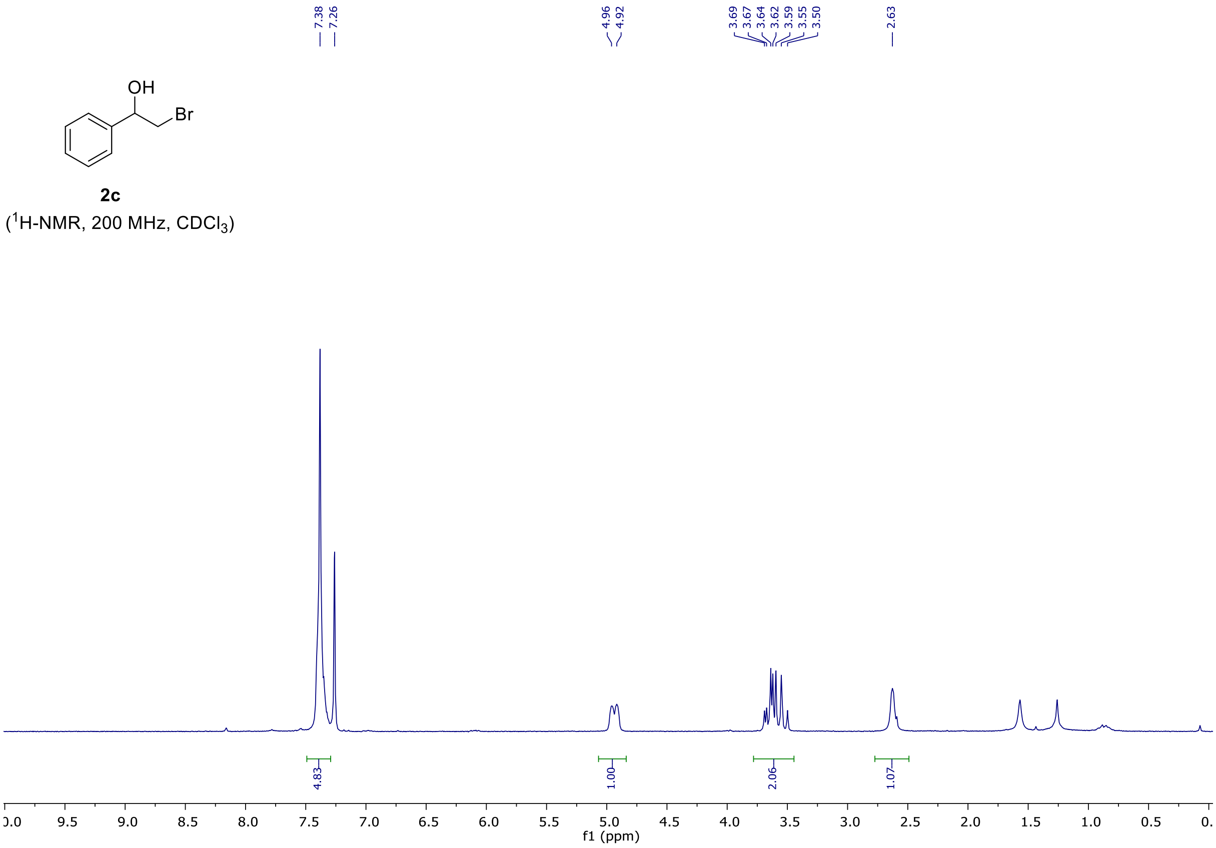
7.38
7.26
4.96
4.92
3.69
3.67
3.64
3.62
3.59
3.55
3.50
2.63
OH
Br
2c
(^{1}H-NMR, 200 MHz, $CDCl_3$)
4.83
1.00
2.06
1.07
f1 (ppm)

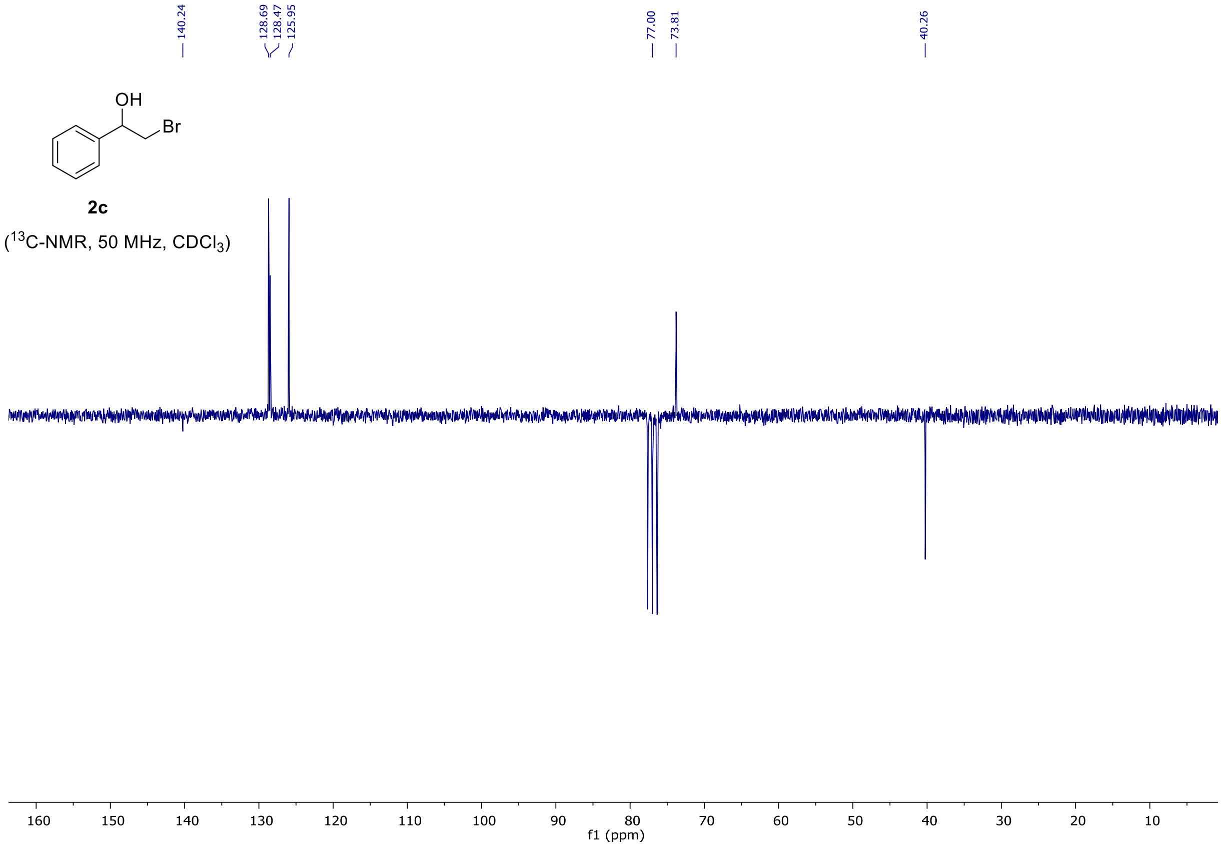
140.24
128.69
128.47
125.95
77.00
73.81
40.26
OH
Br
2c
(^{13}C-NMR, 50 MHz, $CDCl_3$)
f1 (ppm)

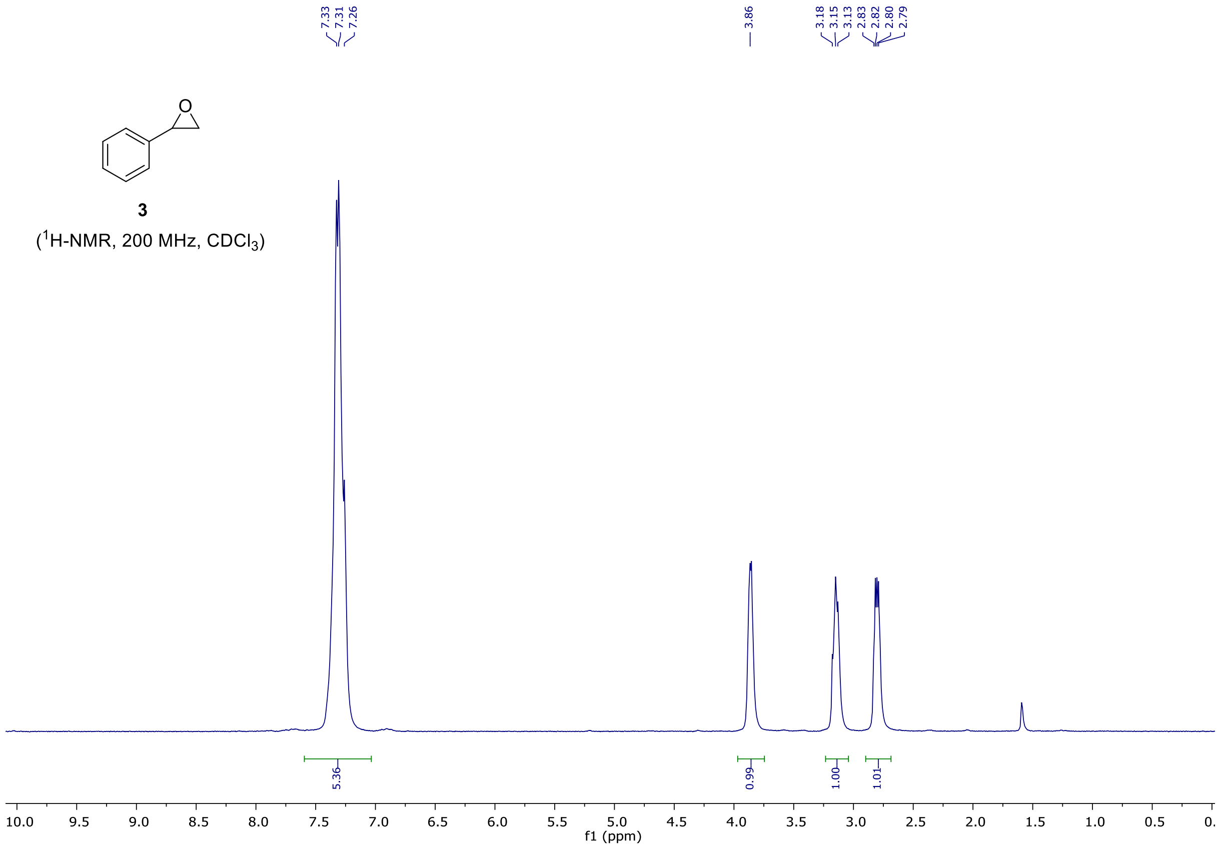
7.33
7.31
7.26
3.86
3.18
3.15
3.13
2.83
2.82
2.80
2.79
3
(^{1}H-NMR, 200 MHz, $CDCl_3$)
5.36
0.99
1.00
1.01
f1 (ppm)

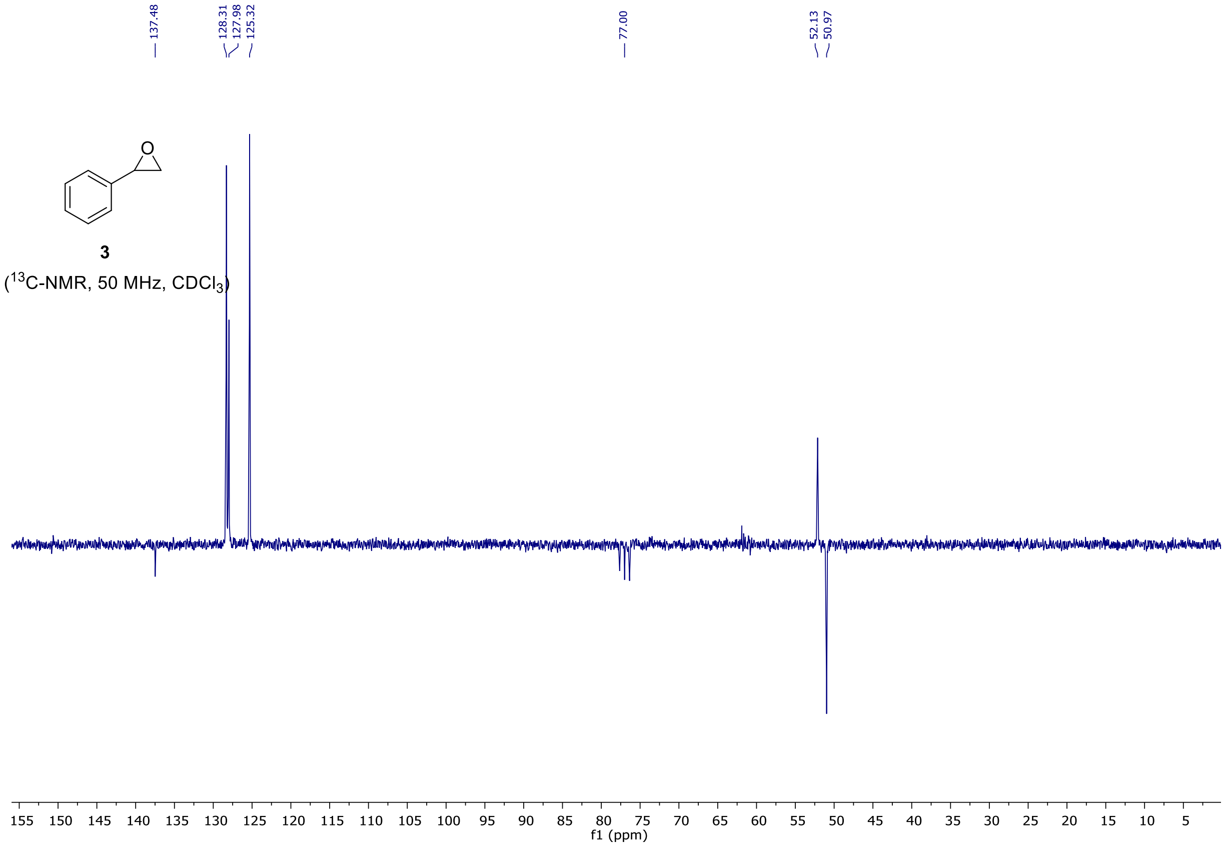
137.48
128.31
127.98
125.32
77.00
52.13
50.97
3
(^{13}C-NMR, 50 MHz, $CDCl_3$)
f1 (ppm)

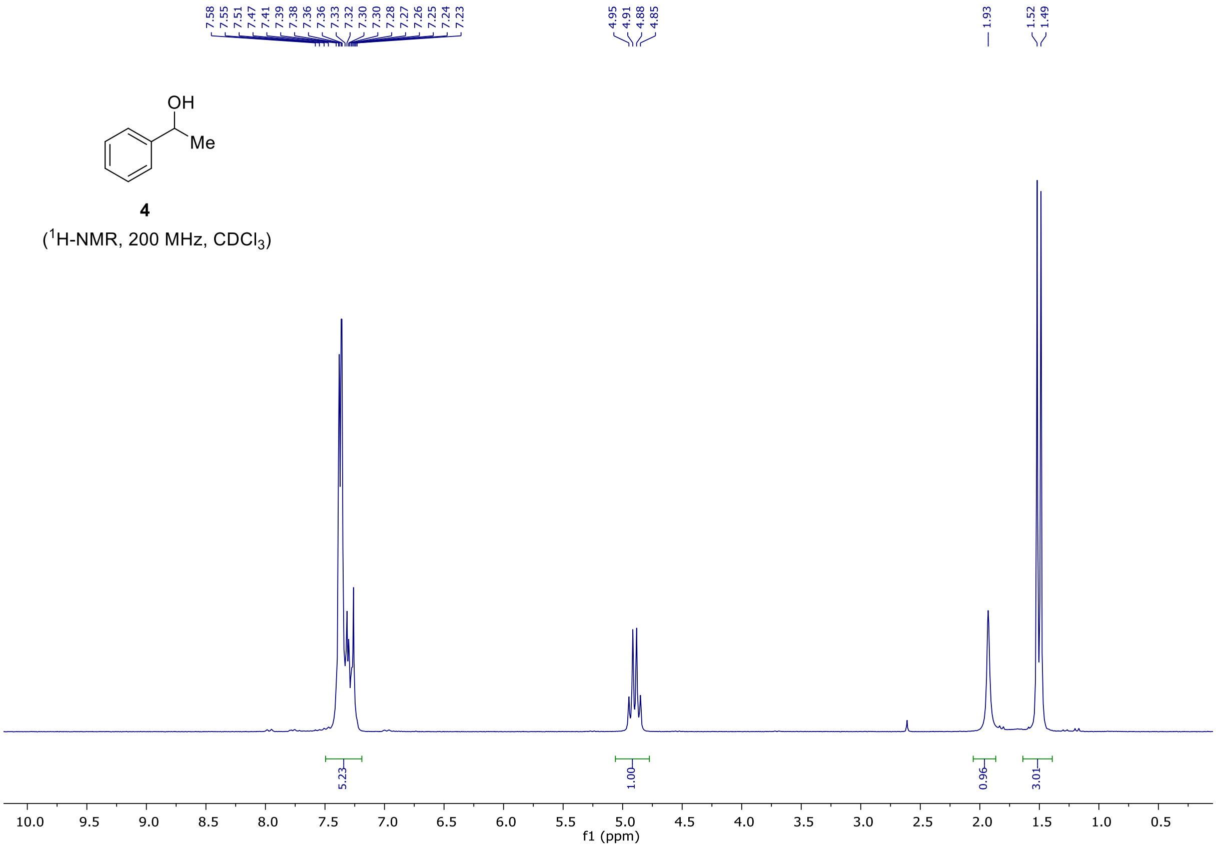

4

(^{1}H-NMR, 200 MHz, $CDCl_3$)

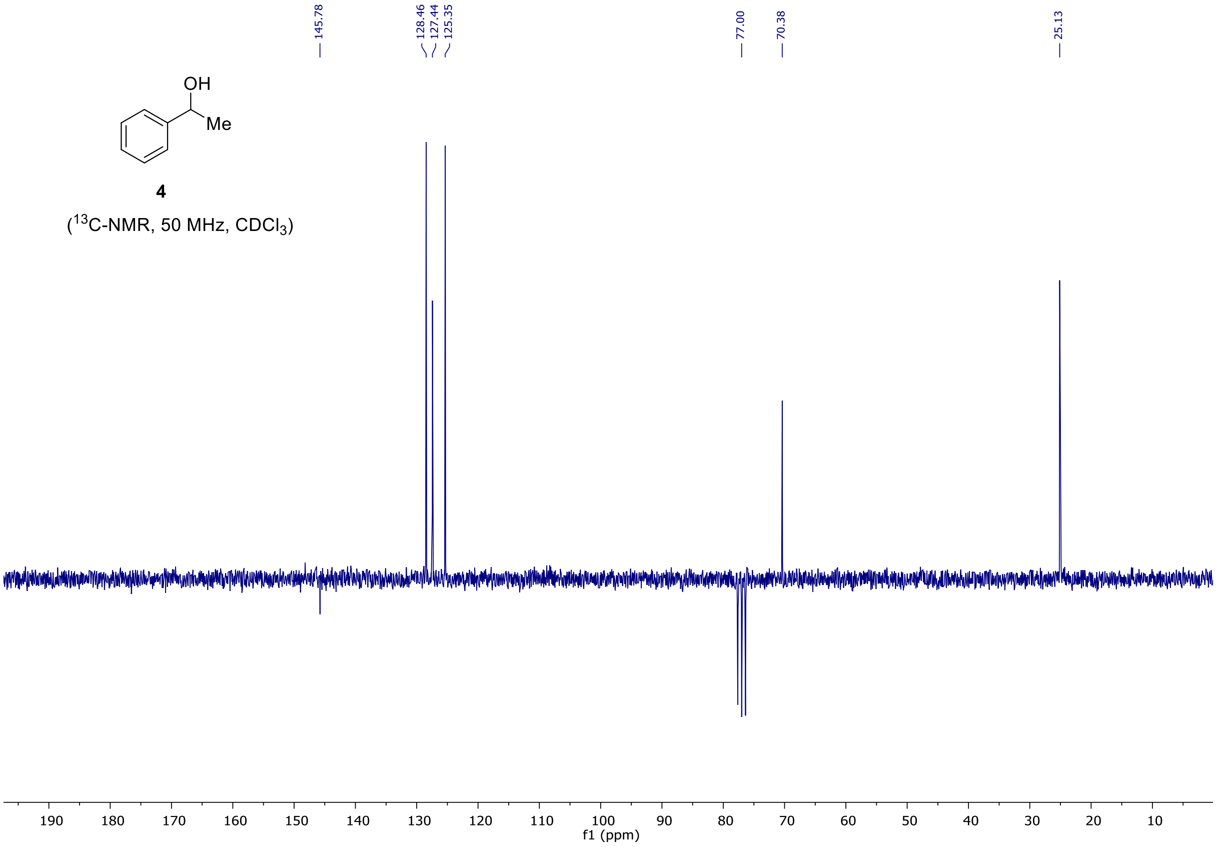

4

(^{13}C-NMR, 50 MHz, $CDCl_3$)

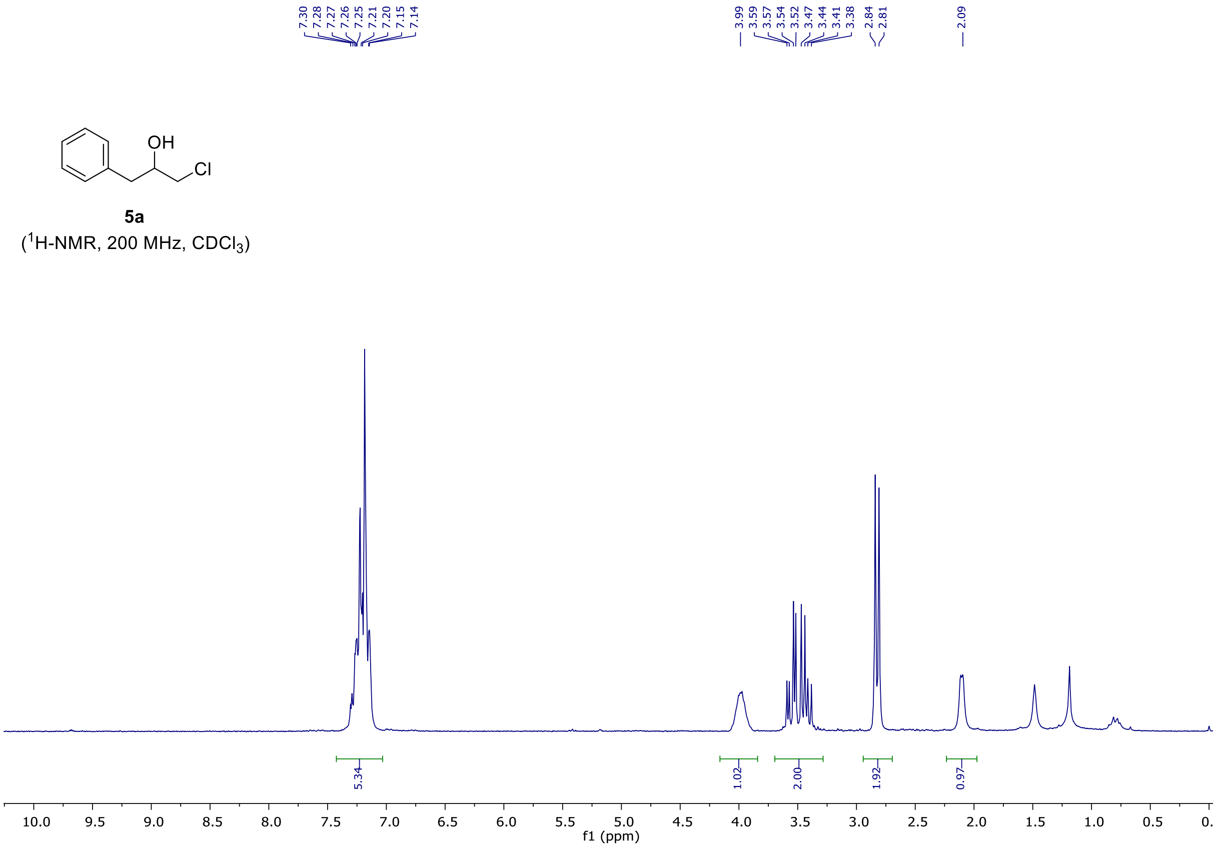

5a

(^{1}H-NMR, 200 MHz, $CDCl_3$)

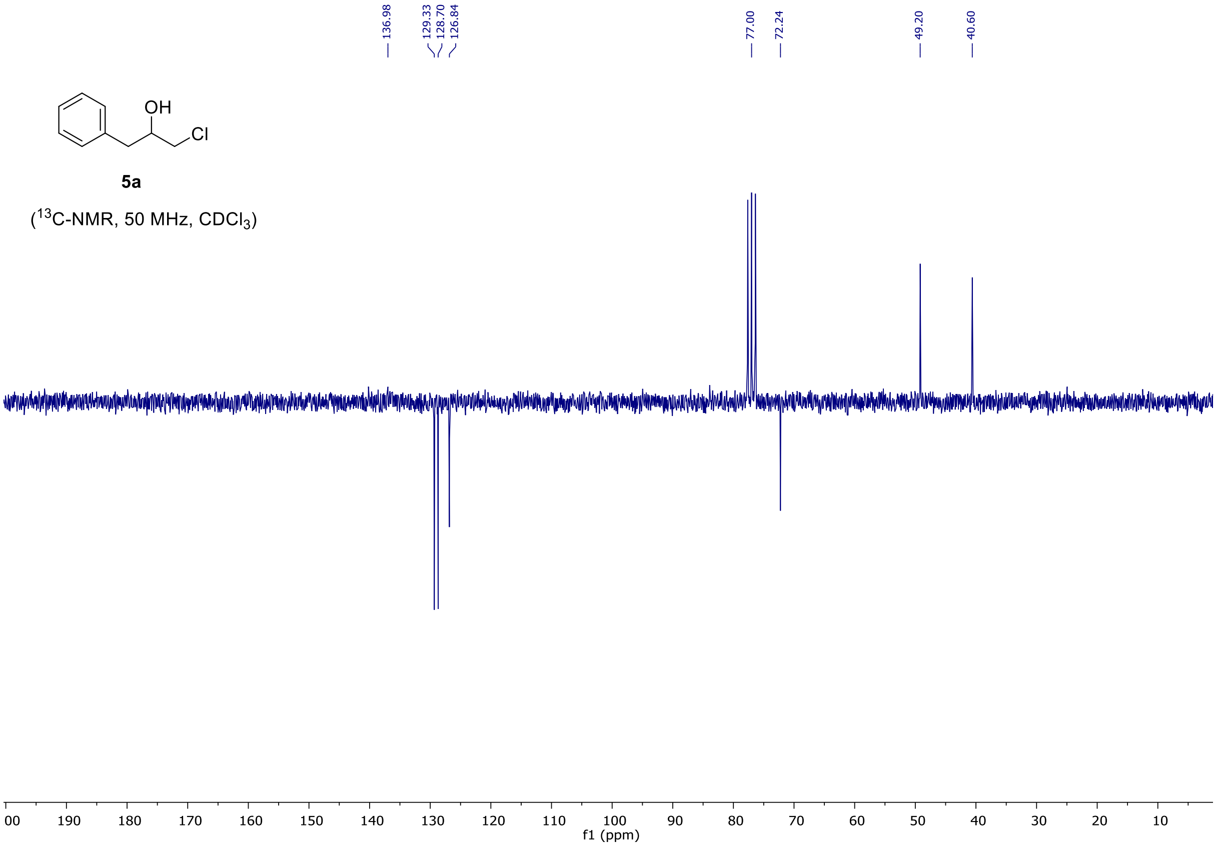

5a

(^{13}C-NMR, 50 MHz, $CDCl_3$)

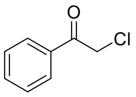

6a

(^{1}H-NMR, 200 MHz, $CDCl_3$)

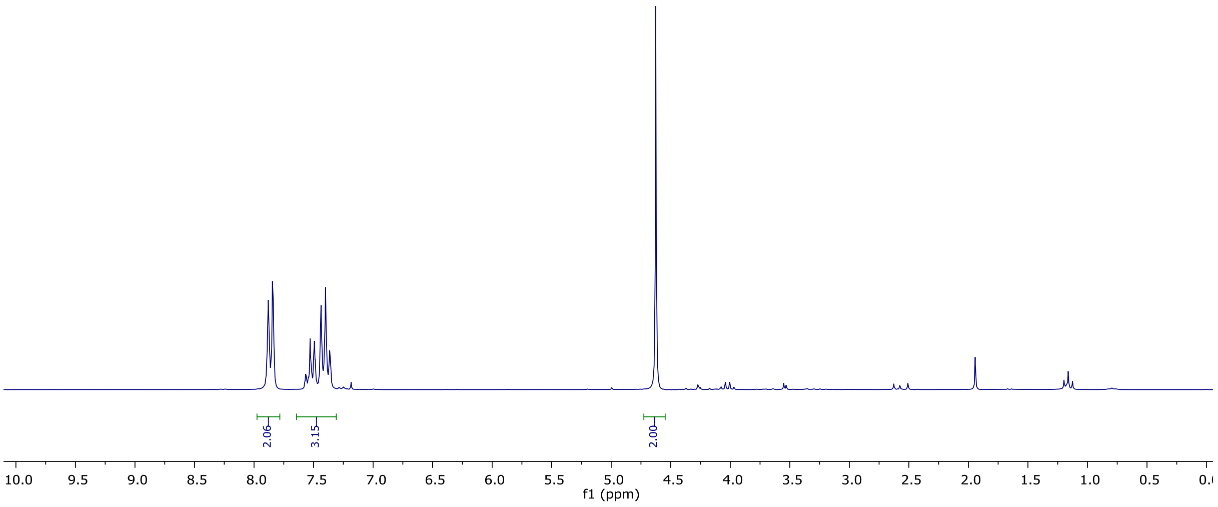

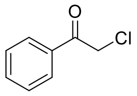

6a

(^{13}C-NMR, 50 MHz, $CDCl_3$)

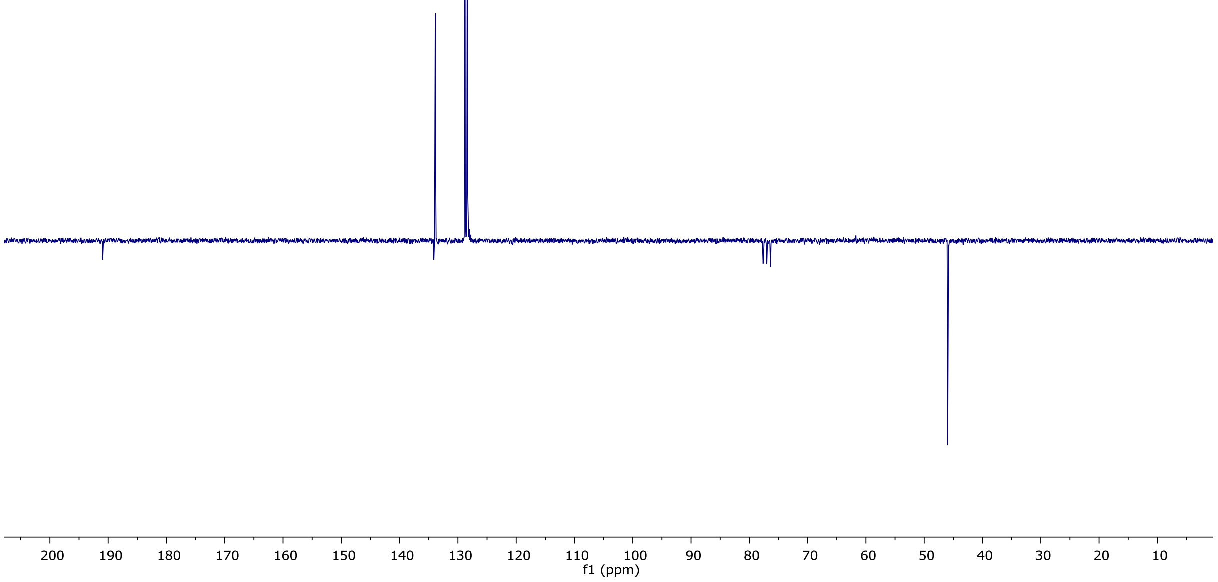

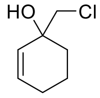

7a

(^{1}H-NMR, 300 MHz, $CDCl_3$)

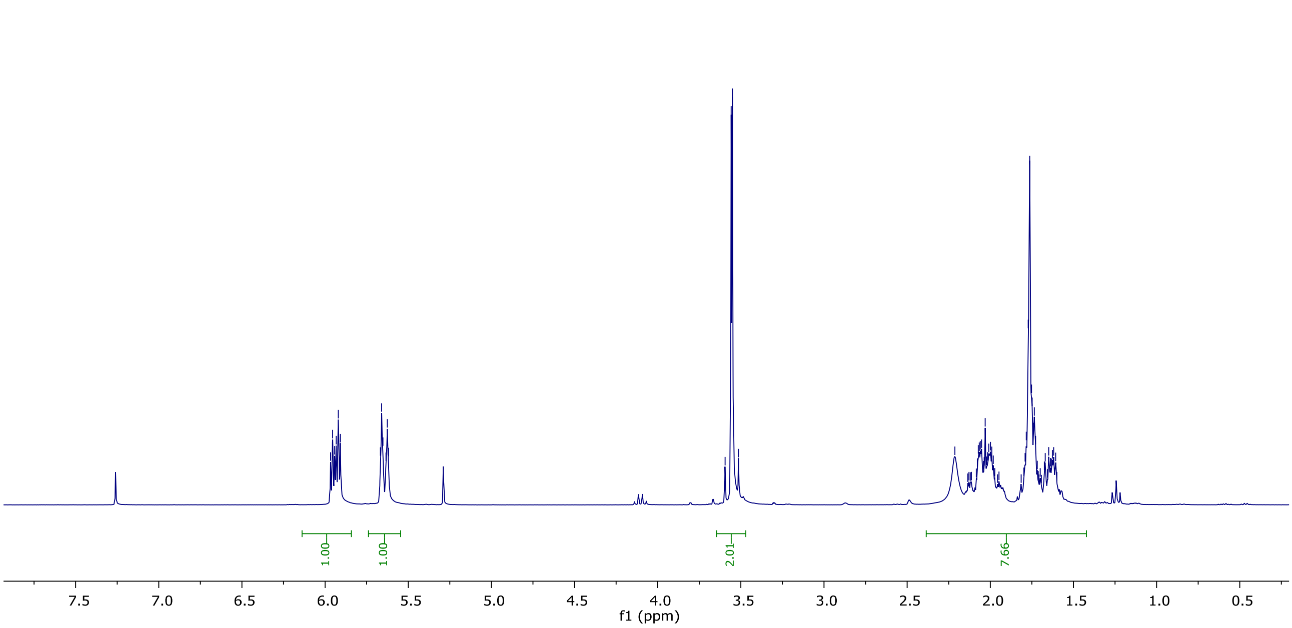

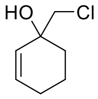

7a

(^{13}C-NMR, 75 MHz, $CDCl_3$)

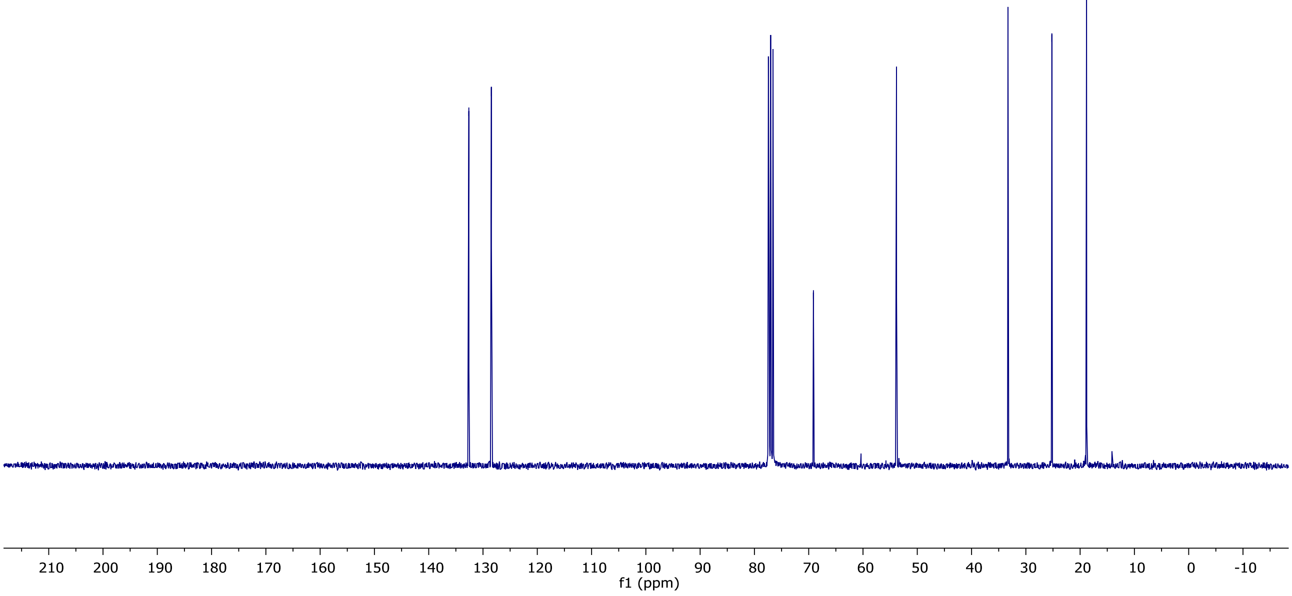

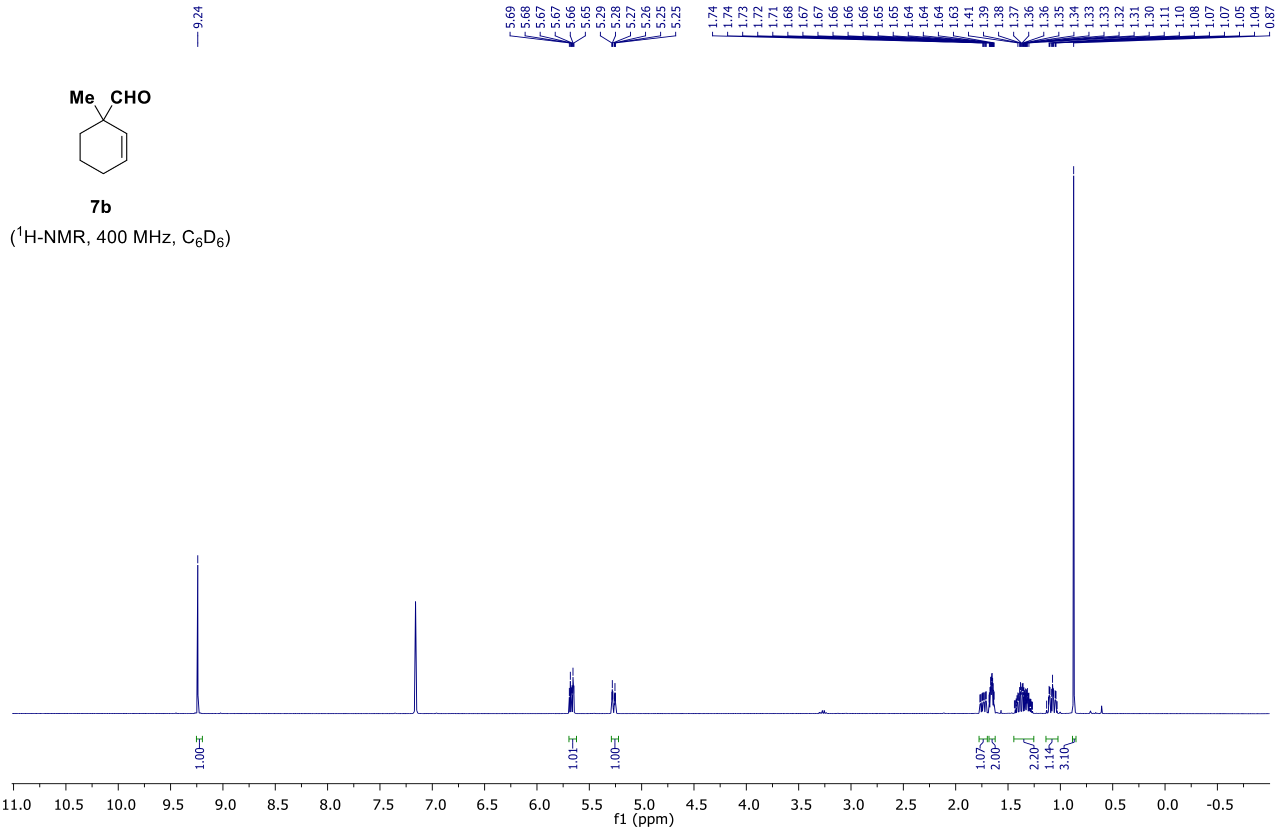
9.24
5.69 5.68 5.67 5.67 5.66 5.65 5.29 5.28 5.27 5.26 5.25 5.25
1.74 1.74 1.73 1.72 1.71 1.68 1.67 1.67 1.66 1.66 1.65 1.65 1.64 1.64 1.64 1.63 1.41 1.39 1.38 1.37 1.36 1.36 1.35 1.34 1.33 1.33 1.32 1.31 1.30 1.11 1.10 1.08 1.07 1.07 1.05 1.04 0.87
Me CHO
7b
(^{1}H-NMR, 400 MHz, C_6D_6)
1.00 1.01 1.00 1.07 2.00 2.20 1.14 3.10
11.0 10.5 10.0 9.5 9.0 8.5 8.0 7.5 7.0 6.5 6.0 5.5 5.0 4.5 4.0 3.5 3.0 2.5 2.0 1.5 1.0 0.5 0.0 -0.5
f1 (ppm)

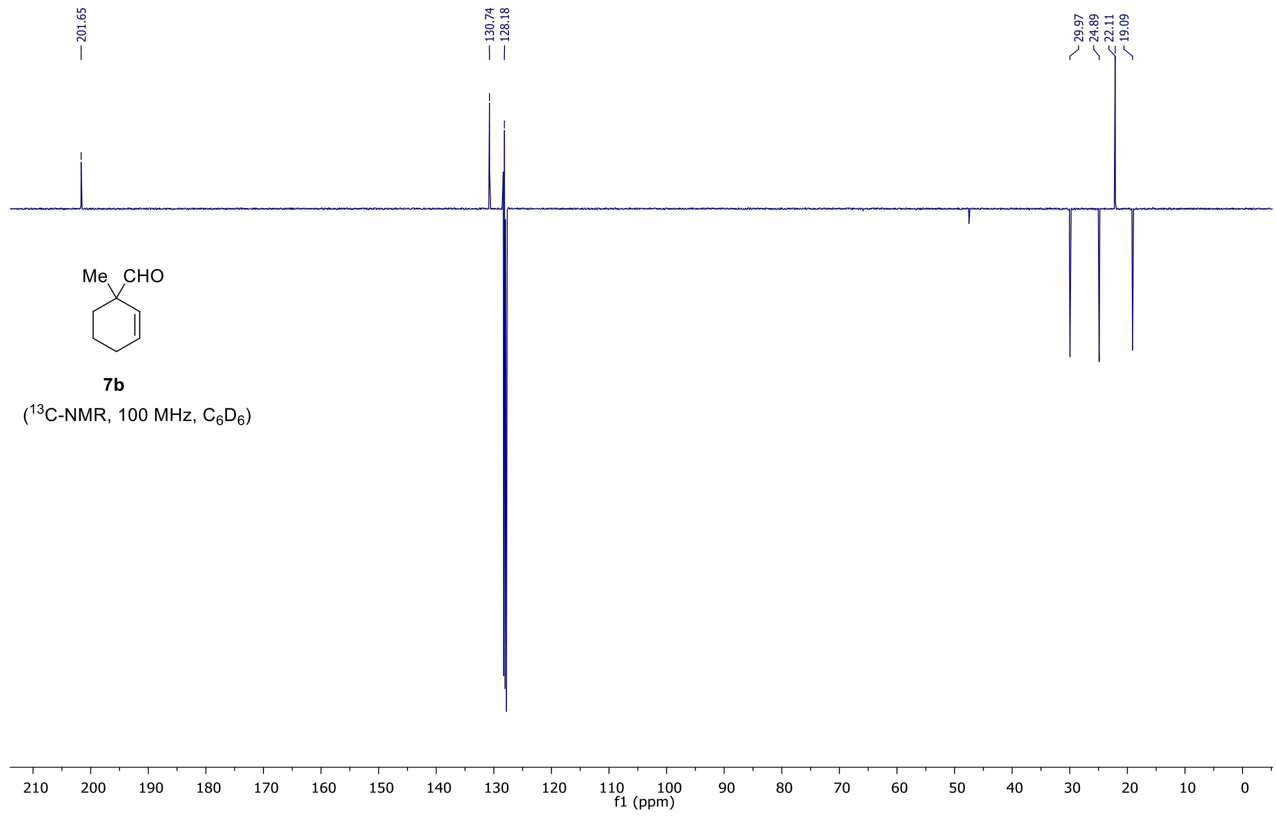
201.65
130.74 128.18
29.97 24.89 22.11 19.09
Me CHO
7b
(^{13}C-NMR, 100 MHz, C_6D_6)
210 200 190 180 170 160 150 140 130 120 110 100 90 80 70 60 50 40 30 20 10 0
f1 (ppm)
